# From Viral Infection to Genome Reshaping: The Triggering Role of HPV Integration in Cervical Cancer

**DOI:** 10.3390/ijms26189214

**Published:** 2025-09-21

**Authors:** Junlan Li, Shuang Li

**Affiliations:** Department of Obstetrics and Gynecology, Tongji Hospital, Tongji Medical College, Huazhong University of Science and Technology, Wuhan 430030, China; m202376483@hust.edu.cn

**Keywords:** HPV integration, cervical carcinogenesis, genomic instability, microhomology-mediated repair, viral–host interaction

## Abstract

Human papillomavirus (HPV) integration is recognized as a hallmark event in cervical carcinogenesis. However, it does not represent a routine phase of the viral life cycle but rather a stochastic occurrence, often constituting a dead-end pathway for the virus. High-risk human papillomavirus (hr-HPV) exhibits a greater propensity for integration. The progression from initial infection to genomic integration constitutes a dynamic multi-step oncogenic process in the development of cervical cancer (CC). This process involves viral entry, immune evasion, persistent infection, and ultimately integration. This article innovatively provides a comprehensive overview of this multi-stage mechanism: HPV, via the L1/L2 proteins, mediates internalization and establishes infection. Subsequently, under the influence of factors such as the host’s genetic background, vaginal microbiota imbalance, and immune evasion, the host’s DNA damage response (DDR) pathways are activated. Viral DNA integrates into host genome vulnerable sites (e.g., 3q28 and 8q24) through microhomology-mediated end joining (MMEJ) or other alternative pathways. Following integration, the expression of viral oncogenes persists, triggering host genomic rearrangements, aberrant epigenetic modifications, and immune microenvironment remodeling, all of which collectively drive cervical cancer progression. The study further reveals the clinical potential of HPV integration as a highly specific molecular biomarker, offering new perspectives for precision screening and targeted therapy. This dynamic model deepens our understanding of the HPV carcinogenic mechanism and provides a theoretical basis for intervention strategies.

## 1. Background

Globally, cervical cancer (CC) ranks as the fourth most common cancer in women, after breast, colorectal, and lung cancers. According to 2022 global cancer statistics, approximately 661,021 new cases and 348,189 deaths occur annually [1]. The incidence and mortality rates vary significantly due to disparities in socioeconomic levels and healthcare services, ranging from 2.2 (1.9–2.4) in Iraq to 84.6 (74.8–94.3) in Eswatini for incidence and from 1.0 (0.8–1.2) in Switzerland to 55.7 (47.7–63.7) in Eswatini for mortality [2]. The World Health Organization (WHO)’s global strategy to eliminate CC aims to reduce its incidence to less than 4 cases per 100,000 women annually within this century [3]. While advancements in prevention and screening technologies have substantially decreased CC rates in high-income countries, low- and middle-income countries (LMICs) still face incidence and mortality rates far exceeding the WHO’s elimination threshold [2]. In most nations, CC is still a considerable public health concern, particularly in sub-Saharan Africa, with advanced treatment posing significant challenges, making it a pressing global public health issue [4].

Nearly all CC cases are linked to persistent infections with high-risk human papillomavirus, such as HPV 16/18 [5]. HPV testing, the primary technique for screening CC, exhibits high sensitivity (>95%) for early infection detection but lower specificity (~80%) [6,7]. Traditional HPV testing relies on viral DNA detection but does not accurately distinguish which HPV infections will progress to CC and which are transient. Furthermore, a limited number of HPV-positive individuals will go on to develop CC—for instance, only 15–20% of infections with HPV16/18 will develop into high-grade squamous intraepithelial lesions (HSILs), but these types pose a much higher risk compared to other oncogenic HPV types [8,9]. Thus, a key challenge in CC prevention lies in effectively triaging HPV-positive individuals to identify high-risk populations for early identification and prevention of CC.

To address the issue, researchers have proposed HPV integration testing as a potential solution [10,11,12]. HPV integration involves inserting the HPV genome into the host’s genetic material, a phenomenon often tied to cervical carcinogenesis. The integration rate of HPV gradually increases from healthy tissue to precancerous abnormalities and eventually to invasive carcinoma [10]. Scientific studies have proven that HPV genome integration is not only related to changes in viral replication capacity but is also closely linked to cellular transformation and tumorigenesis [13]. Consequently, HPV integration testing is expected to serve as a more precise stratification tool, providing critical guidance for further diagnosis and management of high-risk human papillomavirus (hR-HPV)-positive individuals. This review will explore the dynamic process from HPV infection to integration, discuss the role of HPV integration in CC development, analyze its potential as a screening tool, and assess its clinical applicability, aiming to offer a foundational theory for optimizing CC screening strategies.

## 2. Prerequisites for HPV Integration

### 2.1. Viral Factors

HPV consists of a small, circular DNA structure that is double-stranded and non-enveloped with a diameter of approximately 50–60 nm and a genome length of around 8000 base pairs. It exhibits an icosahedral-shaped capsid symmetry. The genome of the virus is split into three sections: the early transcription region, the late transcription region, and the long control region (LCR) or upstream regulatory region (URR). The early region encodes proteins E1 to E8, playing key roles during viral replication, control of transcription, and cancerous transformation. The late region is responsible for encoding the L1 and L2 capsid proteins, involved in viral particle formation and structure. The LCR is the replication origin and contains the transcriptional promoter and enhancers [14].

To date, over 400 HPV genotypes (more than 200 affecting humans) have been identified and classified into five genera: Alpha, Beta, Gamma, Mu, and Nu [15]. Low-risk HPV types primarily cause benign lesions and rarely integrate into the host genome. In contrast, high-risk HPV infection is a major cause of HPV integration into host cells, increasing the risk of carcinogenesis through persistent immune responses and chronic inflammation. According to the WHO, HPVs considered high-risk are types 16, 18, 31, 33, 35, 39, 45, 51, 52, 56, 58, 59, 66, and 68 [16]. HPV integration is detected in over 80% of cervical cancers [17], with HPV16 (clade α9) being the most prevalent oncogenic type, followed by HPV18 and HPV45 (clade α7) [18,19]. Compared to the α9 lineage, the α7 lineage shows a significantly higher integration frequency [20,21], as verified in a comprehensive genomic study of cervical cancer, which found integration across all HPV18 samples and 76% of HPV16 samples [17]. Furthermore, HPV16 is closely associated with squamous cell carcinoma, whereas HPV18 correlates with adenosquamous carcinoma [22], suggesting that different HPV subtypes exhibit different tropisms [17] and molecular differences [23].

Persistent high-copy HPV DNA increases the probability of replication errors, suggesting that viral load increase may enhance the chances of HPV integration [24,25]. In addition, studies show that, during their lifetime, nearly 80% of women will contract HPV, but less than 10% of infected women experience persistent infections [26].

### 2.2. Genetic Susceptibility to CC

Several genetic loci linked to CC risk have been identified through genome-wide association studies (GWASs), particularly inside the major histocompatibility complex (MHC) area. Three independent loci in the MHC were observed to be significantly correlated with CC risk: near MICA, between HLA-DQA1 and HLA-DRB1, and at HLA-DPB2 [27,28]. Furthermore, new susceptibility loci related to HPV infection have been identified through GWASs, including ARRDC3 at 5q14, EXOC1 at 4q12, and CLPTM1L at 17q12 [29,30,31]. These genetic variants may influence CC development by affecting host immune responses, DNA repair mechanisms, and cell cycle regulation, which in turn could impact HPV integration frequency and location [32]. Although candidate gene studies have historically reported numerous associations, many findings suffer from limited reproducibility or inconsistent effect sizes across populations. New understandings of CC pathogenesis have emerged from GWAS and post-GWAS studies, highlighting the complex interplay between genetic factors and HPV infection in disease development [33].

### 2.3. Vaginal Microbiota and Local Immune Environment

The vaginal microbiota in women is diverse, with symbiotic and antagonistic interactions influenced by various internal and external factors, forming a complex vaginal ecosystem. Imbalance in the vaginal microbiota, often termed dysbiosis, plays a critical role in promoting HPV infection and integration [34,35]. Lactobacillus species predominantly make up the normal vaginal microbiota, with a small number of other microbes coexisting; vaginal pH ranges from 3.8 to 4.5. When the vaginal ecosystem is disrupted, changes characterized by abnormal vaginal microbiota and pH occur, primarily due to a reduction in Lactobacillus, leading to pH increase and promoting the proliferation of pathogenic bacteria like *Gardnerella vaginalis*, associated with bacterial vaginosis [36,37]. This shift may trigger chronic inflammation, compromise the cervical epithelial barrier, and facilitate HPV infection [38,39,40]. Additionally, microbial metabolites like butyrate can regulate host gene expression by suppressing histone deacetylase (HDAC) activity, affecting the HPV life cycle [41].

### 2.4. Other Factors

Several factors are tightly associated with HPV infection and CC, including the following: First, nutritional status, as malnutrition can reduce immune function, increasing infection risk [42]. Second, individuals with weakened immune function are more susceptible to HPV, including those with HIV infection, organ transplant recipients, autoimmune disease patients, long-term immunosuppressive drug users, and other immunocompromised patients [43,44].

Additionally, lifestyle habits are significant risk factors, with long-term smoking being an important adjunct factor in CC development. Benzopyrene in tobacco induces APOBEC mutations, increasing the risk of HPV genomic fragmentation [45]. Nicotine suppresses the TLR9 pathway, weakening antiviral immunity [46]. Other factors include estrogen levels, long-term oral contraceptive use, early sexual activity, multiple sexual partners, and multiple pregnancies and births [47].

## 3. Molecular Mechanisms of HPV Entry, Persistent Infection, and Genomic Integration

Following successful nuclear entry, the HPV genome persists as a circular episome [48]. During this stage, viral DNA replicates using the host’s replication machinery while expressing only a limited set of genes, thereby minimizing detectable interference with host cell functions and evading immune surveillance. This phase generally lasts 6–24 months, and most infected individuals clear the virus through immune-mediated mechanisms [9].

A critical transition in HPV infection is the integration of viral DNA into the host genome [49]. This event is particularly common in high-risk HPV types and is a key step toward malignant transformation. Paradoxically, integration also represents a dead end for the viral life cycle. From a clinical perspective, integration marks the transition from infection to carcinogenesis. Cytological abnormalities such as low-grade squamous intraepithelial lesions (LSILs) or HSILs may become detectable at this stage. Integration often occurs during persistent infection (exceeding two years), yet the progression from integration to invasive cancer typically requires additional years or even decades [50], providing a crucial window for clinical intervention. HPV completes the process from infection to genomic integration through a series of precisely regulated molecular mechanisms. There are complex interactions between the virus and host factors in this process.

### 3.1. From HPV Viral Entry to Persistent Infection

#### 3.1.1. Differentiation-Dependent Strategy of the HPV Life Cycle

HPV employs a highly evolved differentiation-dependent replication strategy, wherein its life cycle is intimately associated with the differentiation process of the host’s epithelial cells. Following microtrauma-mediated infection of undifferentiated basal keratinocytes, early viral genes maintain low-copy viral genome replication. As host cells differentiate toward the upper layers, HPV induces the ATM-mediated DNA damage response (DDR) and related pathways to promote viral genome amplification [51,52]. Mature viral particles are ultimately released in large quantities from terminally differentiated keratinocytes [53]. This strategy—localizing persistent infection to basal cells while restricting productive infection to differentiated layers—not only ensures long-term viral infection but also significantly enhances immune evasion.

#### 3.1.2. HPV Infection

**Figure 1:**
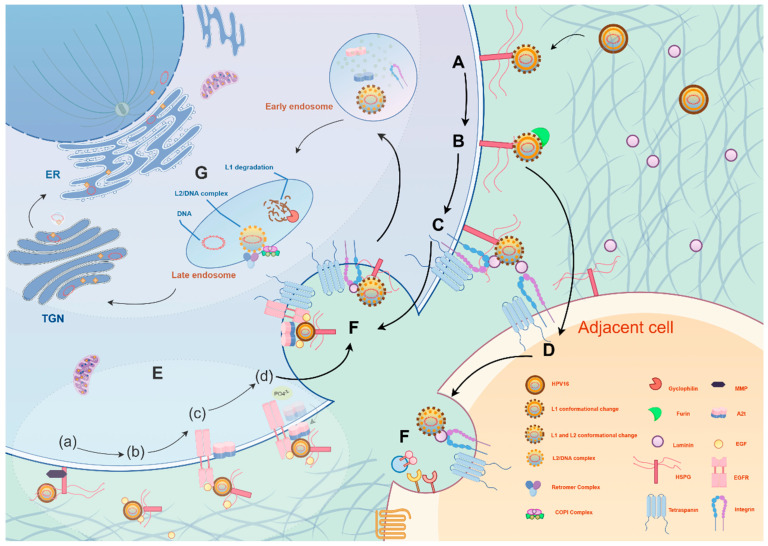
**HPV invades host cells through a multi-step, precisely regulated mechanism**. (**A**) First, the virus L1 protein binds to heparan sulfate proteoglycan (HSPG) on the cell membrane, triggering a conformational change. (**B**) The L2 protein is cleaved by furin protease, exposing the RG-1 epitope, after which the virus enters the cell through two pathways: (**C**) One pathway involves binding to laminin-5 after adsorption, followed by interaction with integrin α6β4, (**D**) or the virus transfers to adjacent cells. (**E**) The second pathway involves the formation of a virus–HSPG–EGF complex, activating the EGFR/Src pathway. (a) The HSPG bound to the virus is cleaved and released by Matrix Metalloproteinase (MMP). (b) The virus, HSPG, and EGF form a high-molecular weight complex. (c) This complex binds to EGFR on the plasma membrane, inducing Src kinase activation. (d) Active Src phosphorylates Annexin A2 (AnxA2), causing AnxA2’s extracellular translocation and binding with the virus. (**F**) The virus is internalized after binding to a four-transmembrane protein. (**G**) The virus is sequentially transported from early endosomes to late endosomes, then to the Trans-Golgi Network (TGN). During this process, L1 is degraded, and L2 cooperates with the Retromer/COPI complex for retrograde transport. Ultimately, the viral genome enters the cell nucleus via mitosis, completing the infection. The figure was made by Figdraw (www.figdraw.com).

At the onset of infection, HPV binds to heparan sulfate proteoglycans (HSPGs) on the surface of host cells via the L1 major capsid protein (Figure 1A) [54]. This initial binding typically occurs on basal keratinocytes. HPV then interacts with laminin-332 (formerly laminin-5) secreted by migrating keratinocytes, serving as a “transient receptor,” facilitating interaction with secondary receptors (such as α6β4 integrins) (Figure 1C), or transferring to adjacent cells expressing secondary receptors (Figure 1D) [55,56]. This process not only enhances virus attachment but also results in structural changes in the capsid, exposing crucial domains of the L2 secondary capsid protein. These changes further facilitate furin protease-mediated cleavage of the L2 protein at the cell membrane, exposing the conserved, cross-neutralizing RG-1 epitope—a critical step for subsequent viral internalization (Figure 1B) [57,58].

The HPV internalization pathway relies on an atypical endocytosis mechanism dependent on actin remodeling and tyrosine kinase signaling (Figure 1E,F) [59,60]. During this process, the C-terminal sequence of L2 can penetrate the endosomal membrane and directly interact with the host cell’s COPI complex, relocating the virus from the endosome to the Golgi apparatus or endoplasmic reticulum [61]. During host cell division, the nuclear membrane temporarily disassembles, and the viral genome, along with L2 protein complexes, enters the cell nucleus (Figure 1G) [62]. Within the nucleus, the HPV genome frequently localizes near nuclear domain 10 (ND10) bodies [63]. These bodies play critical roles in intrinsic immune defense, yet paradoxically provide a favorable niche for viral replication and transcription programs [64]. HPV often reorganizes ND10 components—for instance, the viral L2 protein displaces Sp100 and recruits Daxx, thereby establishing a localized microenvironment conducive to viral transcription and replication initiation [65].

#### 3.1.3. Transcriptional and Replicational Regulation of HPV

The initial phase of HPV infection is characterized via the expression of E1 and E2 proteins, which cooperatively initiate viral DNA replication [66]. E2, the master transcriptional regulator, modulates early promoter activity via conserved E2 binding sites (E2BSs) [67] and recruits the E1 helicase to the replication origin, forming a sequence-specific complex that triggers a cascade of replication events [68,69]. E1, an ATP-dependent helicase, unwinds viral DNA [69] and recruits host replication factors while simultaneously inducing cellular DNA damage responses to promote viral replication [70,71]. Notably, the E2-bromodomain-containing protein 4 (E2-BRD4) complex is known to tether the viral genome to host chromatin for persistent replication in some HPV types; this mechanism exhibits type-specific variation [72]. For instance, HPV16 primarily relies on TopBP1 for chromatin attachment during mitosis, as demonstrated by its essential role in plasmid segregation and retention [73]. Additionally, the viral E8^E2 protein engages with cellular transcriptional repressor factors, suppressing transcription and replication to maintain low-level infection [74,75]. In the higher differentiated layers, viral DNA amplifies to high copy numbers, and L1/L2 capsid proteins autonomously form complete virions. The natural shedding of surface keratinocytes completes the viral dissemination cycle.

#### 3.1.4. Immune Evasion Strategy

The life cycle of HPV, which depends on differentiation, is central to its immune evasion strategy: viral replication remains confined to epithelial cells, avoiding inflammation triggered by cell lysis; high viral loads are restricted to terminally differentiated layers, which are immunologically privileged and destined for desquamation. HPV evades innate immune surveillance by trafficking within endosomal vesicles [76], while the L2 protein counteracts the antiviral effects of PML nuclear bodies [77,78]. Furthermore, viral proteins disrupt the body’s innate immune signaling pathways and postpone adaptive immune reactions, thereby sustaining persistent infection [79]. Notably, the E5 oncoprotein of hr-HPV mediates immune evasion through multiple mechanisms to sustain persistent infection [80]: it primarily sequesters MHC-I/II and CD1d molecules within the endoplasmic reticulum and Golgi apparatus, disrupting antigen presentation and impairing T and NK cell responses. Concurrently, E5 activates the EGF-R signaling axis, upregulating COX-2/PGE_2_ and NF-κB pathways to foster an immunosuppressive microenvironment [81]. This process is accompanied by inactivation of the TGF-β/SMAD tumor-suppressive pathway, further suppressing NK cell cytotoxicity and interferon-mediated antiviral activity [80].

### 3.2. HPV Integration Process

HPV integration is recognized as a hallmark event in cervical carcinogenesis. Integration results in the loss of the virus’s normal replication ability, figuratively described as a “dead end” for replication [82]. Despite the loss of normal replication, this event induces sustained upregulation of E6/E7 oncogenes by disrupting the E2 gene, thereby indirectly potentiating the oncogenic potential.

#### 3.2.1. Disruption of Viral Genome and Activation of Viral Oncogenes

Viral genome integration is a pivotal event in HPV-associated malignant transformation, often accompanied by disruption of *E2/E1* genes [83,84]. The hinge region of *E2* is a frequent breakpoint [83]. Loss of E2 function derepresses the *E6/E7* oncogenes, as E2 normally suppresses their transcription by regulating the early promoter and polyadenylation signal (PAS). Concurrently, truncation of the E1 C-terminal domain impairs its synergy with E2, further exacerbating *E6/E7* overexpression [85,86]. Notably, some studies indicate that the E5 segment is often deleted in integrated HPV clinical samples. This may stem from its genomic location at replication fork convergence points [87]. Following E5 loss, a cascade of events—from single-cell signaling dysregulation to population-level integration—is exponentially accelerated. Specifically, E5 deletion activates the TGF-β/IFN-κ axis, leading to increased secretion of IFN-κ. As a secretory cytokine, IFN-κ diffuses into the microenvironment, promoting clearance of cells harboring episomal viral genomes. Although integrated cells maintain high IFN-κ expression, the viral genome—sheltered within host chromosomes—evades immune eradication, thereby facilitating survival and clonal expansion of integrated clones. This mechanism suggests that E5 loss is not stochastic but rather a necessity under immune micro-pressure, sacrificing local immunosuppressive function (via E5 deletion) to secure long-term advantages conferred by genomic integration. These findings provide a novel paradigm for understanding the evolutionary trajectory of HPV-associated cancers.

These events culminate in the overexpression of the *E6* and *E7* oncogenes. The E6 and E7 proteins drive tumorigenesis through multiple pathways: E6 mediates p53 ubiquitination and degradation via E6AP, inhibiting apoptosis and DNA repair while activating telomerase and disrupting cell polarity [88,89,90]; E7 binds Rb to release E2F, inducing cell cycle dysregulation and generating replicative stress, double-strand breaks (DSBs), and other severe genomic lesions [91,92,93]. Moreover, E6/E7 promotes chronic inflammation via the COX-2/PGE2 axis [94], and the E5 protein amplifies inflammatory responses through the EGFR/NF-κB pathway [81]. The resulting reactive oxygen and nitrogen species (ROS/RNS) induce DNA damage, facilitating viral integration into the host genome [95].

#### 3.2.2. HPV Integration Mechanisms

Integrating the HPV genome into host DNA is a complex, multi-mechanism process centered on the repair-mediated fusion of viral DNA with breakpoints in the host genome. The process initiates with the formation of host genome DSBs, which may be induced by viral proteins [96] or cellular stress responses [97]. Subsequent steps are driven by the DDR, primarily involving four molecular mechanisms: microhomology-mediated end joining (MMEJ), non-homologous end joining (NHEJ), fork stalling and template switching (FoSTeS), and microhomology-mediated break-induced replication (MMBIR).

**Figure 2:**
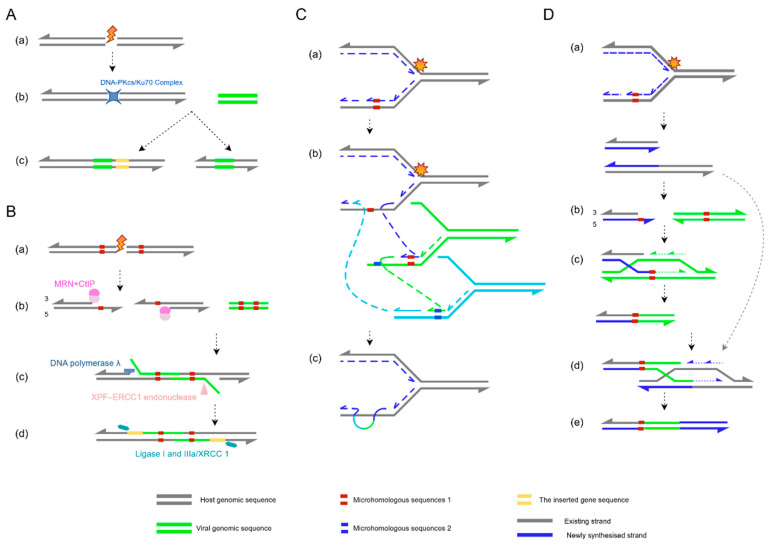
Schematic overview of HPV integration mechanisms. (**A**) Non-homologous end joining (NHEJ): (**a**) A double-strand break (DSB) occurs in the host genome; (**b**) the DNA-protein kinase catalytic subunit (DNA-PKcs)/Ku70 complex directly binds broken ends without requiring homologous sequences; (**c**) ligation often introduces insertions or deletions at the junction site. (**B**) Microhomology-mediated end joining (MMEJ): (**a**) Host genomic damage induces a DSB; (**b**) the Mre11-Rad50-Nbs1(MRN) complex and retinoblastoma binding protein 8(CtIP) cooperatively resect 5′→3′ ends, exposing 3′ microhomology regions; (**c**) microhomologous sequences in HPV DNA pair with host microhomology regions; the excision repair cross-complementing rodent repair deficiency, complementation group 4–excision repair cross-complementing rodent repair deficiency, complementation group 1 (XPF-ERCC1) endonuclease trims non-complementary overhangs, followed by gap filling by DNA polymerase λ; (**d**) DNA ligase I and IIIa/ X-ray complementing defective repair in Chinese hamster cells 1 (XRCC1) ligate the DNA backbone, completing viral integration. This process frequently causes host sequence deletions or rearrangements. (**C**) Fork stalling and template switching (FoSTeS): (**a**) During host DNA replication, the fork stalls at damaged sites; (**b**) the stalled fork switches templates to nearby homologous sequences (e.g., HPV DNA, green); (**c**) HPV DNA is copied into the host genome via template switching, generating complex rearrangements or repeats. (**D**) Microhomology-mediated break-induced replication (MMBIR): (**a**) Host DNA breakage or fork collapse produces a single-ended DSB; (**b**) 5′→3′ resection exposes microhomology regions (red) and creates a 3′ single-stranded overhang; (**c**) the 3′ overhang invades HPV microhomology regions, priming DNA synthesis; (**d**) after that, the strand disengages and reanneals to the original template; (**e**) host–HPV chimeric molecules form, driving genomic rearrangements or HPV integration. The figure was made by Figdraw (www.figdraw.com).

Early studies proposed NHEJ as the dominant pathway for HPV integration [98], given its reliance on the DNA-protein kinase catalytic subunit (DNA-PKcs)/Ku70 complex for erroneous ligation of DNA ends, which can lead to random viral insertion into critical host genes or regulatory regions, triggering genomic instability and oncogenic risk [99] (Figure 2A). However, emerging evidence reveals widespread enrichment of microhomologous sequences at HPV integration sites, suggesting that MMEJ may instead be the predominant mechanism [100].

Initially regarded as a backup repair pathway—sometimes termed alternative NHEJ (alt-NHEJ)—MMEJ can be upregulated upon NHEJ impairment. Yet growing evidence indicates that it may serve as a preferred repair mechanism, particularly in contexts of homologous recombination (HR) deficiency [101]. Recent HPV integration site capture studies confirm that microhomology-mediated repair is the primary mechanism for viral integration [102]. This pathway begins with 5′-to-3′ DNA end resection by the Mre11-Rad50-Nbs1 (MRN) complex in coordination with retinoblastoma binding protein 8 (CtIP), exposing microhomologous sequences at 3′ overhangs [103,104]. After annealing of these microhomology regions, non-complementary flaps are trimmed by the excision repair cross-complementing rodent repair deficiency, complementation group 4–excision repair cross-complementing rodent repair deficiency, complementation group 1(XPF-ERCC1) endonuclease complex [105], gaps are filled by DNA polymerase λ [106], and ligation is completed by DNA ligase I or III [107] (Figure 2B).

Beyond classical repair pathways, HPV integration can also occur via replication-dependent mechanisms like FoSTeS and MMBIR, which generate complex genomic rearrangements [108]. During DNA replication, fork stalling caused by damage or topological stress may trigger template switching, often resulting in interchromosomal rearrangements (Figure 2C). Conversely, replication fork collapse can activate MMBIR—a variant of break-induced replication (BIR) that relies on microhomology rather than long homologous sequences. Unlike canonical Rad51-dependent BIR, MMBIR employs Rad52 to catalyze annealing of short microhomologies [109,110]. Notably, stress conditions such as hypoxia reduce Rad51 levels, promoting MMBIR as an alternative repair mechanism [111]. MMBIR involves 5′ strand resection at DSBs to generate 3′ single-stranded overhangs, which invade microhomology regions of alternative DNA templates to form D-loops and restart synthesis (Figure 2D).

Recent studies propose that microhomology-mediated repair (MMR) generates looping viral–host DNA intermediates, which are amplified through aberrant activation of viral replication origins, ultimately producing head-to-tail concatemers of viral and host DNA [112]. The intricate rearrangement patterns formed by these collaborative mechanisms are a hallmark of HPV-associated cancer genomes.

## 4. Distribution Characteristics of HPV Integration Sites

Several studies have investigated the distribution of HPV integration sites in cell lines and clinical samples. Earlier research suggested that HPV integration sites were randomly distributed on chromosomes [113,114]. However, deeper studies proposed different views, indicating that HPV integration events are not entirely random but show some bias. The concept of “productive integration” has been introduced to explain this phenomenon. Productive integration refers to the virus retaining key oncogenes (E6/E7) and continuously expressing them, directly promoting tumorigenesis. Some studies believe that integration sites are randomly distributed initially, with most being silenced, while productive integration is non-random and closely associated with immune evasion and tumor progression [115].

Different HPV types exhibit different integration tendencies on chromosomal bands, such as HPV16 being prone to forming integration clusters on chromosomes 3 (3q28) and 13 (13q22.1), while HPV18 is more likely to integrate on chromosome 8 (8q24.21) [116]. These regions are rich in cancer-associated genes such as MYC, FHIT, KLF5 and MACROD2, considered HPV integration “hotspots” [117,118,119]. Different histological types of CC exhibit heterogeneity in HPV integration sites, such as adenocarcinoma commonly showing integration at 17q12, while squamous carcinoma exhibits more integration events at 21p11.2 [120]. Additionally, new integration hotspots, such as 14q32.2, 10p15, 2q37, 2q22.3, 3p14.2, 8q24.22, 14q24.1, 17p11.1, 17q23.1, and 17q23.2, have been discovered in recent genome-wide studies, providing new clues for understanding the molecular mechanisms of CC [85,121].

Regarding the distribution of genomic elements, HPV often integrates at common fragile sites (CFSs), transcriptionally active regions, CpG regions, and enhancer regions [49,113,117,122,123]. CFSs are regions on chromosomes susceptible to replication stress-induced breaks. Studies have shown that integration sites are often enriched in FANCD2-associated fragile sites and enhancer-rich regions, with FANCD2 being a protein involved in DNA repair. This enrichment may contribute to genomic instability, further promoting cancer development [49].

## 5. Trigger Effects of HPV Integration

HPV integration types are typically classified into single-copy integration, multi-copy integration, and mixed types. A recent study discovered a new type of integration lacking E6/E7 genes, suggesting that although E6/E7 oncogenes are key contributors to the development of cancer, they are not the only mechanism, highlighting the diversity and complexity of cancer progression mechanisms [124]. The HPV genome’s integration acts like a “trigger” for CC development, initiating a series of complex chain reactions. This process not only alters the virus’s own genome structure but also causes a chain of transformations in the host genome, including the induction of host genome instability and transformations in host gene expression regulation. This virus–host genome interaction plays a crucial role in the occurrence and progression of CC, further promoting cancer development.

Notably, although viral genomic integration is a key event in carcinogenesis, it is not an absolute requirement for malignant transformation. Approximately 15% of cervical carcinomas have been found to retain HPV in a purely episomal form, demonstrating that integration is not an obligatory step in oncogenesis [125]. Specifically, episomal HPV achieves carcinogenesis through dynamic epigenetic mechanisms—such as increased acetylation of histone H4 within the upstream regulatory region (URR), particularly at late promoter regions. This not only drives early transcriptional activation of the E6/E7 oncogenes but also compensates for later transcriptional downregulation by maintaining high viral copy numbers, thereby ensuring sustained oncoprotein expression. Remarkably, this process exhibits fundamental similarities to the integration pathway in in vitro models: both depend on E6/E7-mediated degradation of p53 and pRb to promote uncontrolled proliferation and share immune evasion strategies (e.g., downregulation of interferon-stimulated genes). Furthermore, episomal forms of HPV16 can form polymers and mutants through abnormal replication and rearrangement, ultimately leading to cancerous transformation [126]. These findings provide a mechanistic basis for the episome as an independent oncogenic pathway.

Recent studies have established that oropharyngeal cancer has now surpassed cervical cancer as the most common HPV-associated malignancy. In the United States and other developed countries, approximately 70% of oropharyngeal cancer cases are HPV-positive [127]. Dynamic epigenetic regulation may explain the enhanced sensitivity of these tumors to radiotherapy and chemotherapy (e.g., reversible histone modifications) [128], offering a rationale for HDAC-targeted therapeutic strategies.

Furthermore, the coexistence of episomal and integrated HPV copies acts as an amplifier during carcinogenesis—a phenomenon elucidated in studies such as that by Kadaja et al. [129]. When both forms are present in a cell, E1 and E2 proteins expressed from episomal HPV activate replication origins at integrated sites, triggering “onion-skin replication”. This process generates aberrant replication intermediates, including linear DNA, branched structures, and circular plasmids, directly causing double-strand breaks (DSBs). Moreover, these lesions recruit cellular repair machinery, which often fails due to overloaded capacity, subsequently activating the ATM-Chk2 pathway and ultimately inducing irreversible chromosomal abnormalities such as translocations [129].

### 5.1. HPV Integration Leads to Host Genome Instability

Studies show that HPV integration directly promotes genomic instability [112], leading to focal structural alterations, generating virus–host fusion transcripts, forming super-enhancers, and other alterations in the host genome. These genomic changes, in turn, exacerbate genomic instability, forming a vicious cycle.

#### 5.1.1. Chromosomal Structural Variations or Chromosomal Rearrangements

In a study of HPV-CCDC106 integration sites, HPV integration significantly altered the local chromosomal three-dimensional structure [130]. Whole-genome sequencing (WGS), transcriptome sequencing, chromatin immunoprecipitation (ChIP) sequencing, and high-throughput chromosome conformation capture (Hi-C) analysis of fresh tumor tissues revealed significant genomic variations and differential expression features on chromosome 19 [130]. HPV integration split a topologically associating domain (TAD) into two smaller TADs and hijacked an enhancer element from the PEG3 gene to the CCDC106 site, leading to PEG3 repression and CCDC106 upregulation [130].

Further research has shown that HPV integration can trigger structural and numerical variations in chromosomes [131]. Studies on HPV16 integration have observed that chromosomal translocation events occur simultaneously with HPV integration, suggesting that this process may lead to chromosomal instability [132]. Virus integration disrupts telomere stability or interferes with centromere function, leading to mitotic abnormalities [133]. Additionally, HPV-16 E6 and E7 proteins cooperate to disrupt centrosome duplication, promoting the formation of multipolar spindles and further exacerbating aneuploidy [134,135].

The breakage–fusion–bridge (BFB) cycle, a key genomic rearrangement mechanism in cell lines, can lead to complex inversions and gene amplification events on chromosomes. For example, on chromosome 11, genes such as YAP1, BIRC2, and BIRC3 frequently undergo amplification through the BFB cycle, and these genetic alterations are significantly associated with early CC diagnosis and higher invasiveness [136]. Furthermore, recent studies reveal that, following integration into the host genome, HPV forms virus–host heterocateny—complex concatemeric structures exhibiting high instability. These complexes mediate the capture, amplification, and rearrangement of host genomic segments through dynamic excision and reintegration processes [137]. This mechanism underlies the diverse and interconnected genomic rearrangement patterns observed within individual tumors, substantially promoting intratumoral heterogeneity and clonal evolution.

#### 5.1.2. Virus–Host Fusion Transcripts

Studies of HPV16- and HPV18-related CCs and precancerous lesions have found that HPV integration can induce the production of virus–host fusion transcripts, which is reflected in genomic analyses of CCs: 83% of HPV-positive tumors exhibited virus–host fusion transcripts [17]. These transcripts may use the host’s PAS, affecting the expression of viral oncogenes [138]. Research shows that despite multiple integrated copies of viral DNA, the virus–host fusion transcripts typically originate from a single integrated HPV DNA sequence, and host genome elements are crucial for the effective expression of viral oncogenes. This aberrant expression may lead to abnormal proliferation and carcinogenesis [139]. Furthermore, virus–host fusion transcripts are more stable than those produced by free viruses, possibly because integrated HPV lacks the unstable core motif “AUUUA” in its 3’ UTR [140].

#### 5.1.3. Virus–Cell Super-Enhancers

HPV integration sites frequently appear at transcriptional regulatory hubs, typically associated with cell-specific enhancers. Studies have shown that HPV16 integration can hijack and polymerize cell enhancers to generate virus–cell super-enhancers (SEs). In studies of CC cell lines, approximately 26 copies of HPV16 integrated into the intergenic region of chromosome 2p23.2, interspersed with amplified flanking cell DNA, with this region being rich in super-enhancer markers such as H3K27ac and Brd4. The hybrid elements formed by this integration can significantly upregulate the expression of E6/E7 oncogenes [141].

Notably, HPV-derived SEs can also function through extrachromosomal DNA (ecDNA). By conducting a multi-omics analysis of six HPV-positive and three HPV-negative cell lines, seven high-activity HPV breakpoint-induced cellular super-enhancers (BP-cSEs) were identified. These elements are located on HPV–human hybrid ecDNA and mediate transcriptional activation through long-distance intra- and interchromosomal interactions. This process regulates the expression of multiple target genes, and pathway analysis confirms their enrichment in carcinogenic networks [142]. These findings reveal a new mechanism by which HPV integration forms SEs through chromosomal and extrachromosomal mechanisms, providing new insights into viral carcinogenesis.

### 5.2. Downstream Effects of HPV Integration-Induced Oncoprotein Dysregulation

#### 5.2.1. Epigenetic Modifications

HPV genome integration can lead to widespread changes in both viral and host epigenetic features [143], promoting immune evasion and tumorigenesis. Allele-specific differentially methylated regions (DMRs) near integration sites can span megabases, independently of transcriptional status, suggesting widespread epigenetic dysregulation as a direct consequence of HPV integration—a finding corroborated by recent nanopore sequencing data [144]. Some studies have found that high methylation of immune-stimulating CpG sites in the E6/E7 region of integrated HPV16 weakens TLR9 recognition [145]; meanwhile, methylation of URR and E2BSs disrupts the transcriptional repressive function of E2 on viral oncogenes [146,147,148,149]. Furthermore, methylation of the L1/L2 regions further promotes immune evasion and persistent infection [150]. A multi-omics analysis of 50 CC cases found that HPV integration near host genes such as MIR205HG and PROS1 alters their methylation and expression patterns. Specifically, integration at the MIR205HG enhancer region upregulates its expression, while high methylation in the PROS1 promoter region silences its expression [150].

Importantly, E6/E7 can exacerbate genomic instability and promote malignant transformation by regulating epigenetic modification factors such as HDAC, DNMT3B [151], and RNA-binding proteins such as RNASEH2A [152]. These findings confirm the critical importance of HPV integration in the course of epigenetic reprogramming of CC and supply important evidence for the development of novel biomarkers and therapeutic targets.

#### 5.2.2. Somatic Mutations

HPV integration can induce mutations in the host genome through various mechanisms. Typical mechanisms include the following: (1) Direct induction of mutations by disrupting host genome integrity. For instance, HPV integration commonly targets the regulatory regions of tumor-suppressor genes (such as TP53 and SCAI) or proto-oncogenes (such as PIK3CA and ERBB2) [132,153], promoting carcinogenesis through insertion mutations or promoter activation. (2) Disrupting DNA repair systems (such as RAD51B and FBXW7 mutations), exacerbating genomic instability and leading to mutation accumulation [153]. These mutations are often enriched near HPV integration sites and are linked to the advancement of tumors. Studies show that mutations in genes like LRP1B in HPV16-integrated CCs are significantly associated with poor prognosis [153,154], indicating the key role of integration-associated mutations in disease progression.

#### 5.2.3. Copy Number Variations

HPV integration can induce large-scale copy number variations (CNVs) in the host genome [133,155]. The integration process is accompanied by the formation of DSBs, which activate NHEJ or MMR [132], leading to the amplification or deletion of local genes. For example, HPV16 integration into the 3q28 chromosomal region containing the PIK3CA gene can trigger amplification of this region, activating the PI3K/Akt/mTOR signaling pathway [156]. Studies show that the upregulation of MYC and HMGA2 oncogenes is closely associated with HPV integration in their flanking regions [100]. Additionally, the amplification of genes such as CD274 (PD-L1) and PDCD1LG2 (PD-L2) suggests a link between CNVs and immune therapy response [17]. Comparative genomic hybridization (CGH) analysis reveals that the amplification of 1q, 3q, and 20q, along with the deletion of 11q and 13q, is considerably elevated in HPV-positive cervical tumors compared to HPV-negative ones, revealing the existence of integration-specific CNV patterns [157,158].

### 5.3. Virus-Mediated Immune Microenvironment Remodeling

Comprehensive analysis of single-cell RNA sequencing (scRNA-seq) and spatial transcriptomics (ST) has uncovered a dynamic “homeostasis–imbalance–malignancy” transition in the immune microenvironment during CC development [159]. In early stages, immune cells effectively eliminate tumor cells to maintain homeostasis. However, as the disease progresses, the accumulation of immunosuppressive cells (e.g., Tregs and M2-type tumor-associated macrophages [TAMs]) drives immune evasion, facilitating tumor infection and metastasis [160,161]. HPV oncoproteins play a pivotal role in this process. They impair antigen presentation by suppressing dendritic cell function, downregulate immune surveillance molecules such as TLR9 [162,163], disrupt interferon synthesis, and promote the secretion of immunosuppressive factors, thereby polarizing the immune microenvironment and fostering immune escape. Viral genome integration further exacerbates immune evasion through PD-L1-mediated T cell exhaustion and HLA loss, creating conditions for persistent viral infection and malignant transformation [164,165]. In advanced stages, the immune microenvironment deteriorates further, a process characterized by heightened immunosuppression and functional exhaustion of immune cells. During this phase, tumor cells engage in complex crosstalk with immune cells to promote infection and metastasis [166]. For instance, PCLAF+ tumor-associated epithelial cells (TAEpis) suppress CD8+ T cell function, supporting tumor growth [161].

## 6. Clinical Translation: From Triage to Targeted Therapy

HPV integration holds potential application value in CC triage screening. Compared to cytological examination triage, HPV integration shows similar sensitivity and negative predictive value for detecting CIN3+ lesions, but with higher specificity, and a significantly lower referral rate for colposcopy [10,167]. Therefore, combining HPV integration detection may optimize triage strategies for high-risk patients, enabling more effective screening of high-risk individuals and reducing unnecessary diagnostic and therapeutic procedures.

Additionally, targeted therapeutic strategies for HPV integration mainly include inhibiting DNA repair mechanisms and viral oncogene expression. HPV integration induces genomic instability via MMEJ, and inhibiting MMEJ-related repair enzymes such as poly(ADP-ribose) polymerase (PARP) may effectively block the integration process [168]. Another strategy is to target the expression of the E6 and E7 genes, which interfere with the cell cycle [91]. For example, using small interfering RNA (siRNA) technology can effectively reduce the expression levels of E6 and E7, thereby enhancing the effectiveness of chemotherapy drugs [169]. Furthermore, gene editing technologies like CRISPR/Cas9 could precisely repair the genomic abnormalities caused by HPV integration, offering a potential therapeutic approach [170]. Meanwhile, inhibiting ecDNA excision and recombination—key steps in heterocateny—may disrupt clonal evolution [137], presenting a promising strategy for HPV-associated cancers. While these strategies are still in the exploratory stage, future research is expected to provide new options for the treatment of CC.

## 7. Conclusions

HPV integration represents a pivotal event in carcinogenesis, yet its accidental nature and the existence of episome-driven oncogenic pathways must be acknowledged. The process from HPV infection to integration is a dynamic multi-stage process involving complex interactions between the virus and host cells. In the initial stage, HPV enters the host cell via L1/L2 proteins, establishing initial infection. Later, under the combined influence of host genetic background and ecological imbalance, the viral genome integrates into the human chromosome via mechanisms such as MMEJ and NHEJ. This process plays a pivotal role in CC development, triggering a cascade of events. HPV integration not only directly induces localized genomic disruption—including DNA breakage, cellular super-enhancer formation, and virus–host fusion transcripts—but also indirectly amplifies genomic instability through sustained E6/E7 expression, which promotes the accumulation of somatic mutations, CNVs, epigenetic modifications, and activation of immune escape mechanisms. High-throughput technologies have revolutionized HPV integration research. Techniques like WGS [133,171], scRNA-seq [139], long-read sequencing [124], and the Hi-C technique [102] allow researchers to capture specific integration sites, frequencies, and their genomic impacts with high precision and efficiency.

Future research directions should focus on elucidating the integration mechanisms of different HPV subtypes and their specific impacts on the host genome, in particular how distinct integration patterns influence the clinical manifestations and prognosis of CC. Additionally, investigations into the distribution and functional consequences of HPV integration across different tissue types—especially the differences in integration patterns between adenocarcinoma and squamous cell carcinoma—are warranted. A deeper understanding of the molecular mechanisms underlying HPV integration, as well as potential alternative pathways, remains crucial. Concurrently, the mechanisms driving carcinogenesis in HPV-negative patients deserve further attention.

Collectively, the dynamic process from HPV infection to integration not only reveals the complex interplay between the virus and host cells but also provides a theoretical foundation for early diagnosis and targeted therapy of CC. Moving forward, targeted interventions against HPV integration will emerge as pivotal strategies in cancer screening and treatment, offering novel clinical approaches. Supported by high-throughput technologies, research in this field will accelerate our understanding of HPV-mediated oncogenesis and enhance the precision of early diagnosis and therapeutic interventions.

## Data Availability

Not applicable.
